# A moderated mediation analysis of occupational stress, presenteeism, and turnover intention among occupational therapists in Korea

**DOI:** 10.1002/1348-9585.12153

**Published:** 2020-07-27

**Authors:** Byung‐Yoon Chun, Chiang‐Soon Song

**Affiliations:** ^1^ Department of Tax & Management College of Management Gwangju University Gwangju Korea; ^2^ Department of Occupational Therapy College of Health Science Chosun University Gwangju Korea

**Keywords:** moderated‐mediation effect, occupational stress, perceived organizational support, presenteeism, turnover intention

## Abstract

**Objectives:**

Presenteeism is undoubtedly a widespread phenomenon in organizations. Research on presenteeism has been conducted for decades in the broader workforce (eg, nurses, doctors, teachers, police officers). Occupational stress and turnover intention in occupational therapy have been extensively studied. However, the effect of presenteeism on the relationship between occupational stress and resultant turnover intention among occupational therapists is unclear. This study aims to explore the mediating effect of presenteeism and moderating effect of perceived organizational support in the relationship between occupational stress and turnover intention among occupational therapists in Korea.

**Methods:**

We conducted an individual and cross‐sectional analysis of 257 occupational therapists from various health care institutions in Korea. Data were collected and hypotheses were tested via Process macro. Quantitative analyses were conducted with SPSS 26 and LISREL 8.54.

**Results:**

Occupational stress was strongly related to presenteeism, which in turn predicted turnover intention. Presenteeism played a mediating role between occupational stress and turnover intention. Moreover, occupational therapists’ perception of organizational support acted as an important mechanism through which presenteeism mediated the relationship between occupational stress and turnover intention.

**Conclusions:**

This study highlights the need to maximize employee productivity and retain talent by providing managers with insight into the mechanism of presenteeism in relation to occupational stress and turnover intention among occupational therapists in Korea.

## 1 INTRODUCTION

Maintaining high productivity and retaining talent are vital for organizational competitive advantage. However, the recent global economic downturn and skyrocketing unemployment rate have led to radical changes in the work environment, which increased demands with less resources and organizational support.^1^ According to the Health Care Workforce Survey 2018 conducted in Korea,^2^ 14 727 occupational therapists (OTs) possess a license, of which 77% of them were female. Furthermore, only 44.9% of the licensed OTs were on active duty, working for various health care institutions. Assuming the current status remains constant in Korea, OTs are likely to be in a surplus between 3100 and 4150 or up to 22% more than the demand since 2020 onward, in contrast to doctors and nurses who are expected to be scarce in number.^3^ Due to change in the economic and labor market environment, organizations tend to implement cost containment strategies,^4^ which creates a lack of job security among employees. Therefore, employees are likely to refrain from behaviors that are socially sanctioned and reluctantly show up to work even while feeling sick in order to project a positive attitude at work.^5^

An instance where an employee attends work despite feeling ill instead of taking a leave for rest is described as “presenteeism”.^6^ It takes place when employees physically appear at work, but are unable to function effectively.^7^ In such cases employees are likely to be less productive, make more mistakes, provide lower‐quality service, and be less innovative.^8^ Once regarded as a good behavior and an antonym of absenteeism, presenteeism is now considered as negative and avoidable as it reduces individual and organizational productivity.^9^ Thus, managing presenteeism in organizations would offer greater opportunities for advancing competitive advantage.^10^

Presenteeism among care and education professionals such as nurses, physicians, and teachers has been extensively studied,^6^ but has received only limited attention in occupational therapy. Presenteeism arises in professional and highly skilled white‐collar jobs,^11^ in jobs where clients heavily rely on services (eg, doctors, teachers, government workers), and where no backup resources are available (eg, scheduled duty).^6^ Therefore, OTs are also likely to engage in presenteeism owing to patients’ reliance on care and unavailability of replacements. Moreover, Korean OTs may be more likely to engage in presenteeism considering the culturally dominant traits of valuing hard work and perseverance.^5^

Johns (2010) developed a model which defines the prevalence of presenteeism in terms of personal, contextual, and health‐related factors.^7^ Although the majority of research on presenteeism has focused on health problems,^12^ limited attention has been granted to presenteeism generated by stress.

The term *stress* is conceptually divergent and there is a lack of consensus on its definition.^13^ An appropriate amount of stress boosts creativity in performing tasks and provides motivation to improve productivity at the workplace,^14^ but an excessive amount of stress may lead to negative performance.^13^ As per the Job Demand‐Resources (JD‐R) theory, each occupation has its own specific risk factors (ie, job demands and job resources) associated with job‐related stress.^15^ Although OTs are part of a multidisciplinary professional team, they often work alone. Most OTs have become used to performing their professional care under demand due to a lack of available backup.^16^ This is more obvious in the sizeable minority of health care institutions in Korea. OTs in Korea are also highly stressed due to the heterogeneous composition of members, the dual‐command structures of medical and administrative functions, and increasingly more disputes and competition among health care institutions.^17^ Nevertheless, they go to work, but are functionally out of work due to feeling ill. Therefore, occupational stress (OS) is one of the key drivers of presenteeism.^18^

In addition, employee turnover remains a critical issue for organizations and managers because of the costs involved with recruiting and training newcomers, which is often more than the annual salary for the position being hired for.^19^ Turnover intention tends to be the strongest turnover predictor.^19^ OS has also been implied as a predictor of negative attitudinal and behaviora1 outcomes such as job dissatisfaction, organizational commitment, and intention to leave.^20^

Presenteeism may be seen as an active and problem‐focused coping strategy,^21^ characterized by maximizing effort to overcome work‐related demands. Thus, based on the transactional theory of stress,^22^ employees might continue going to work despite feeling ill because of a sense of loyalty toward their job, colleagues, or clients, or fear of social criticism, or job insecurity when they encounter OS. Adopting the conservation of resources (COR) theory by Hobfoll (1988),^23^ when OTs with OS realize their resources are limited, they may try to preserve and protect their resources (which prompts them to go to work while sick) and even consume energy improving existing resources (which evaluates the present job across alternatives available and evolves intentions about what to achieve). Therefore, we assumed that OTs under OS are likely to go to work while unwell, resulting in an increasingly high propensity of turnover intention. Thus, we formulated the following hypothesis to examine the role of presenteeism in the relationship between perceived OS and turnover intention:

Hypothesis 1

Presenteeism mediates the relationship between OS and turnover intention.

In a recent survey in Korea, 30.2% of 511 OTs reported intentions to leave their current employer within 6 months because they felt they are underpaid, work under poor environments, and have an excessive workload, including work beyond the scope of their job such as helping in administrative work and assisting in preparation of accreditation evaluation for the organization.^2^ Even if the supply exceeds the demand of OT workforce in Korea, hiring and retaining experienced OTs will be equally important. Thus far, the antecedents of employee turnover and turnover intentions have represented a key area of research. Researchers have focused significant attention on the concept of perceived organizational support (POS) as a key predictor of turnover intention.^24^ POS is defined as the extent to which employees believe that the organization values their contribution and cares about their well‐being.^25^ They perceive themselves as being valued when they yield benefits such as approval and respect, pay and promotion, and access to information and other forms of aid needed to better accomplish their job.^26^

POS acts both to lessen directly and buffer against levels of OS^27^ and to affect work outcomes through the cues the company sends regarding the employees’ work contributions.^28^ That is, POS may facilitate successful coping with a stressful situation. POS is also negatively related to turnover intention.^29^ Consistent with organizational support theory,^25^ POS may reduce turnover by strengthening the obligation felt toward the organization and reinforcing employees’ sense of belonging and identification with the organization, thus moderating the relationship between presenteeism and turnover intention. Moreover, the prediction that POS will moderate the relationship between presenteeism and turnover intention implies that POS will conditionally affect the strength of the indirect relationship between OS and turnover intention through presenteeism. Therefore, we assume higher POS would be associated with less turnover intention and conditionally associated with the effect of OS on turnover intention through presenteeism. Thus, the following hypotheses were examined:

Hypothesis 2

The positive relationship between presenteeism and turnover intention is moderated by POS, such that the relationship is weaker when POS is higher.

Hypothesis 3

The indirect effect of OS on turnover intention through presenteeism depends on the level of POS among OTs, such that the mediating effect of presenteeism in the positive relationship between OS and turnover intention is expected to be weaker when POS is higher.

**Figure 1:**
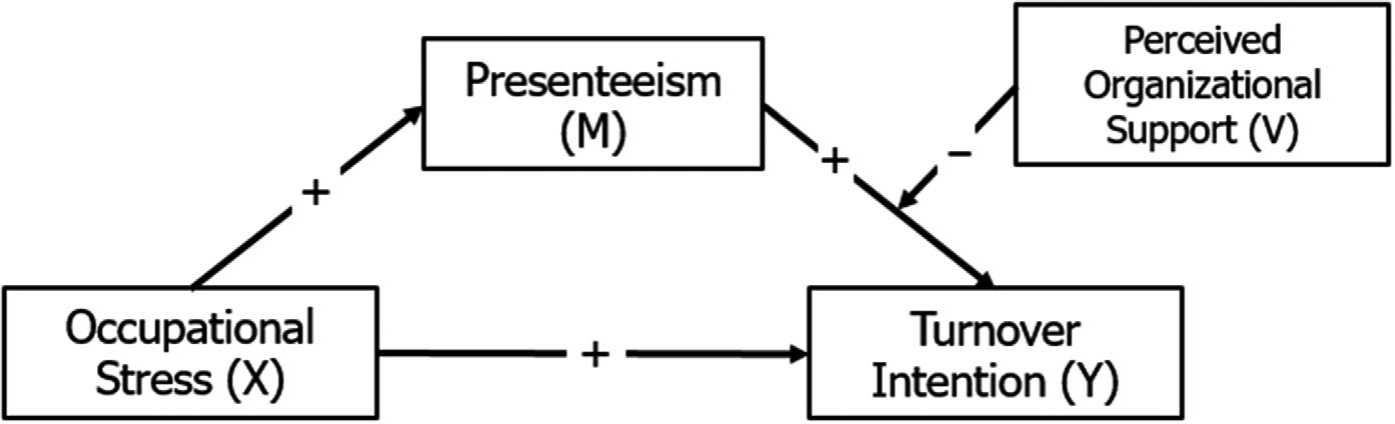
The hypothesized moderated‐mediation model

On the basis of the existing theory and research, the current study aimed to (a) assess the mediating role of presenteeism in the relationship between OS and turnover intention, and (b) investigate the alleviating role of POS in the association between presenteeism and turnover intention. A graphical illustration of the hypothesized relations is reported in Figure 1.

## 2 SUBJECTS AND METHODS

### 2.1 Participants and data collection

We collected data, using a structured questionnaire, from OTs across 23 different health care institutions in Korea, including both publicly and privately owned institutions. We visited each institution to recruit participants and obtained informed consent from all participants prior to data collection. To ensure anonymity, we collected only personal information necessary for the study. Participants were assured that all information provided in the questionnaires would remain confidential. After removing 11 missing responses, we analyzed the data of 257 participants.

### 2.2 Demographic characteristics of the participants

The sample constituted predominantly of women (77.8%), who were in their 20s (77.8%), university graduates (75.9%), and unmarried (80.2%). Among these, 19.8% belonged to double income families and 11.7% had preschool‐age kid(s). Moreover, 82.9% of participants were regular full‐time employees, 47.1% possessed a work experience of 1 to 5 years, 89.9% were general‐duty OTs, and 52.9% worked at privately owned health care institutions.

### 2.3 Measurement tools

We adapted measurement scales with satisfactory validity and reliability coefficients, from previously published studies. As all the tools, except for OS, were originally developed in English, we adopted the translation and back‐translation procedure recommended by Brislin (1986).^30^ All variables, except for demographic variables, were rated on a 5‐point Likert scale ranging from 1 to 5 (“strongly disagree” to “strongly agree”).

We measured the participants’ perception of OS using the Korean Occupational Stress‐Short Form (KOSS‐SF), developed by Chang et al (2005).^31^ The KOSS‐SF contains seven subscales including: job demand (4 items), insufficient job control (4 items), interpersonal conflict (3 items), job insecurity (2 items), organizational system (4 items), a lack of rewards (3 items), and occupational climate (4 items). Higher scores indicate a higher level of OS. Internal consistency was 0.84.

We assessed presenteeism using 10 items of the Stanford Presenteeism Scale (SPS), developed by Turpin et al (2004).^32^ Although the SPS measures the impact of diverse health conditions, we only included items measuring work impairment. Higher scores represent larger presenteeism. Internal consistency was 0.78.

We assessed the participants’ intention to quit using three items developed by Landau and Hammer (1986).^33^ Higher scores indicate greater intention to leave the organization. Internal consistency was 0.83.

We measured the participants’ POS using four items from the shortened version of the Survey of Perceived Organizational Support (SPOS), developed by Eisenberger et al (1986).^25^ We used the four items of the SPOS scales with the highest factor loading indicator in their analysis. Internal consistency was 0.86. Higher scores represent a higher level of POS.

In addition, we controlled three demographic variables—gender, employment type, and tenure. The data regarding gender (dummy coded *female = 1, male = 0*) and employment type (dummy coded *permanent = 1, temporary = 0*) were recorded as dichotomous variables and tenure was transformed to a continuous variable (*such less than 1 year as 0.5, 1 ~ 5 years as 2.5, 5 ~ 10 years as 7.5, 10 ~ 15 years as 12.5, and over 15 years as 17.5*). Moreover, we included perceived health as a control variable due to its associations with our test variables.^34^

### 2.4 Data analyses

Data were analyzed using SPSS (v.26) and LISREL 8.54. We first conducted a descriptive analysis to obtain the means, standard deviations, and frequencies for demographic variables. We then conducted a confirmatory factor analysis (CFA) to test the validity of each variable. Since the data were collected using self‐reported measures, we verified the potential existence of common method variance (CMV) by comparing our research model with Harman's one factor model,^35^ using chi‐square with degree of freedom, root mean square error of approximation (RMSEA), standardized root mean square residual (SRMR), comparative fit index (CFI), normed fit index (NFI), and incremental fit index (IFI). Furthermore, we calculated Cronbach's alpha to test the internal‐consistency for the main variables with a threshold of 0.7 and above.^36^ We also confirmed our model for convergent and discriminant validities using the average variance extracted (AVE) values. Before testing the hypotheses, we performed Pearson's correlations analysis to explore the relationships among the four latent variables including the control variables. Furthermore, we employed the macro program ‘Process’ developed by Preacher and Hayes (2004) for SPSS to test the mediation effect of presenteeism in the relationship between OS and turnover intention and the moderated mediation effect of POS.^37^ We tested the effects on the basis of 5000 bootstrapped samples and BC (bias‐corrected) 95% of confidence intervals (CIs).

## 3 RESULTS

### 3.1 Validity and reliability

We conducted CFA for the key survey items to ensure all variables were distinct constructs. We deleted two items in the subscales of OS (insufficient job control) with factor loading indicators less than 0.4 as recommended by Stevens (2002).^38^

To assess the potential bias of CMV, we tested χ^2^, CFI, NFI, IFI, SRMR, and RMSEA by comparing the research model with Harman's one‐factor model.^35^ Higher values of CFI, NFI, and IFI, and lower values of SRMR and RMSEA are indictors of good fit. The results reported that the research model (χ^2^ = 1174.70, *df* = 647, CFI = 0.95, NFI = 0.90, IFI = 0.95, RMSEA = 0.056, SRMR = 0.062) depicted a better fit than Harman's one‐factor model (χ^2^ = 2987.16, *df* = 702, CFI = 0.80, NFI = 0.75, IFI = 0.80, RMSEA = 0.13, SRMR = 0.11). Thus, there was no significant bias due to CMV in our research model.

We estimated the internal consistency reliability using Cronbach's alphas. The Cronbach's alpha values for OS, presenteeism, turnover intention, and POS were 0.84, 0.78, 0.83, and 0.86, respectively. As the Cronbach's alpha for all the scales was greater than 0.7, as recommended by Nunnally & Bernstein (1994),^36^ all scales depicted internal consistency reliability. Based on the high construct reliability, we tested both convergent and discriminant validity using the AVE values to determine the acceptable levels. Convergent validity is acceptable when the AVE value of each variable exceeds 0.5; discriminant validity is secured, in a further rigorous manner, when the AVE value of each construct is greater than the square of its correlation coefficient with the other constructs.^39^ As the AVE values were 0.506 for OS, 0.561 for presenteeism, 0.653 for turnover intention, and 0.637 for POS, and all the AVEs were larger than the square of their correlation coefficients with other constructs, we were confident of the existence of both convergent and discriminant validity.

### 3.2 Correlations

The correlation coefficients (r) are displayed in Table 1. OS had a significant positive correlation with both presenteeism (0.549, *P* < .001) and turnover intention (0.386, *P* < .001), but a significant negative correlation with POS (−0.674, *P* < .001). Presenteeism showed a significant positive correlation with turnover intention (0.338, *P* < .001) and a significant negative one with POS (−0.489, *P* < .001). POS depicted a significant negative correlation with turnover intention (−0.398, *P* < .001). Among the control variables, female (gender) had a significant positive correlation with OS, presenteeism and turnover intention (0.231, *P* < .001; 0.302, *P* < .001; 0.124, *P* < .05, respectively), and a significant negative correlation with POS (−0.198, *P* < .01). Furthermore, permanent employment and tenure showed positive correlations with presenteeism and OS (0.216, *P* < .01; 0.165, *P* < .01, respectively). Perceived health condition was the only factor that shared significant associations with all four variables of OS (−0.455, *P* < .001), presenteeism (−0.474, *P* < .001), turnover intention (−0.239, *P* < .001), and POS (0.423, *P* < .001).

**Table 1:** Mean, standard deviation, correlations, and Cronbach's alphas among variables (N = 257)

	*M*	SD	*r*							
			(1)	(2)	(3)	(4)	(5)	(6)	(7)	(8)
(1) Gender	0.78	0.42	1							
(2) PE	0.83	0.38	0.056	1						
(3) Tenure	4.35	4.47	−0.034	0.031	1					
(4) PH	3.03	1.02	−0.309***, a	−0.191**	−0.103	1				
(5) OS	2.82	0.47	0.231***, a	0.105	0.165**	−0.455***, a	(0.835)^†^, ^b^			
(6) PRT	3.04	0.57	0.302***, a	0.216**	0.093	−0.474***, a	0.549***, a	(0.784)		
(7) POS	2.85	0.80	−0.198**	−0.187**	−0.053	0.423***, a	−0.674***, a	−0.489***, a	(0.859)	
(8) TOI	2.72	1.02	0.124*	0.005	−0.113	−0.239***, a	0.386***, a	0.338***, a	−0.398***, a	(0.828)

Abbreviations: OS, Occupational stress; PE, Permanent employment; PH, Perceived health; POS, Perceived organizational support; PRT, Presenteeism; TOI, Turnover intention.

* *P* < .05.

** *P* < .01.

*** *P* < .001.

^†^ The numbers in parenthesis indicate Cronbach's alphas.

We conducted a simple mediation analysis and examined the indirect effects via the ‘Process’ program based on bootstrapped CIs. The indirect effect was significant at 95% level of significance, as indicated when the lower and upper levels of the CIs did not contain zeroes as reported in Table 2. The results show that all the key variables, namely, OS, presenteeism, and turnover intention, were significantly and positively related and in the expected directions (OS to presenteeism, 0.4845, *P* < .001; presenteeism to turnover intention, 0.3329, *P* < .05; and OS to turnover intention, 0.6556, *P* < .001). The indirect effect of OS on turnover intention through presenteeism was 0.1613, providing support for Hypothesis 1.

**Table 2:** Simple mediation results (N = 257)

Variables	Model 1		Model 2	
	Presenteeism		Turnover intention	
	B^a^, ^*^	*SE*	B	*SE*
Control variables:				
Gender (female = 1)	0.1811*, ***	0.0716	−0.0509	0.1487
Employment type (permanent = 1)	0.1853*, ***	0.0758	−0.1908	0.1573
Tenure	0.0005	0.0064	−0.0422**	0.0131
Perceived health	−0.1279***	0.0323	−0.0523	0.0684
Independent variables:				
Occupational stress	0.4845***	0.0677	0.6556***	0.1524
Presenteeism			0.3329*, ***	0.1295
*R* ^2^	0.3945***		0.2099***	
Bootstrap indirect effect on turnover intention (through presenteeism)^b^, ^**^	B	*SE*	LL 95% CI	UL 95% CI
OS	0.1613	0.0642	0.0411	0.2952

Abbreviations: CI, confidence interval; LL, lower limit; UL, upper limit.

^a^ Unstandardized coefficients are reported; Direct and total effects.

^b^ Bootstrap sample size = 5000.

* *P* < .05.

** *P* < .01.

*** *P* < .001.

**Figure 2:**
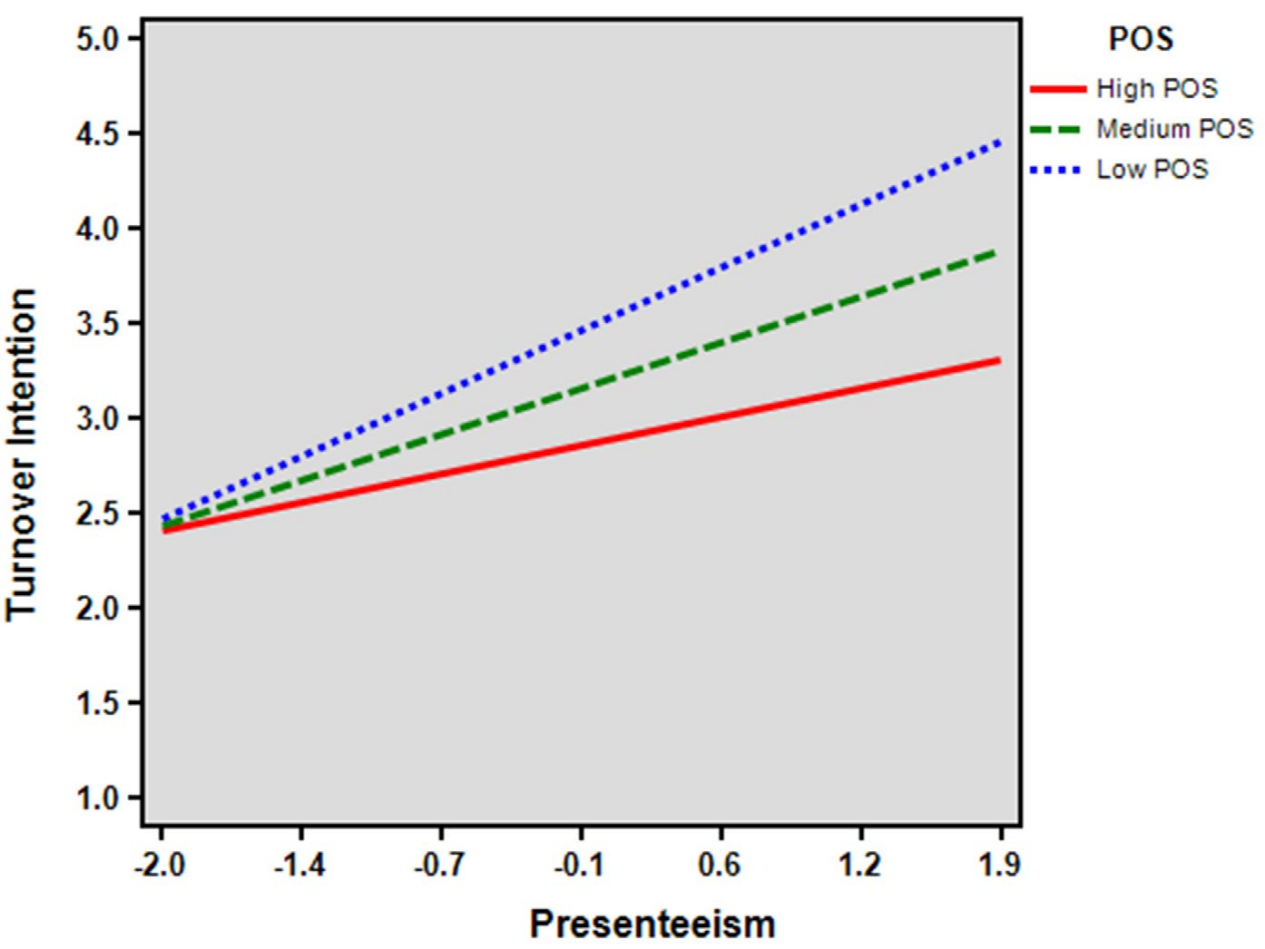
Relationship between presenteeism and turnover intention at values of POS

Table 3 depicts the results of the moderation test predicted in Hypothesis 2 and the moderated‐mediation related to the conditional indirect effect in Hypothesis 3. The results revealed that POS moderated the relationship between presenteeism and turnover intention (−0.1819, *P* < .05). These results support Hypothesis 2. Therefore, OTs with a lower level of POS combined with a higher level of presenteeism reported a higher level of turnover intention. We displayed the relationship graphically in Figure 2.

**Table 3:** Moderated mediation results (N = 257)^***^, ^a^

	To turnover intention (Y)	
	B	SE
Control variables:		
Gender (1 = female)	0.0061	0.1483
Employment type (permanent = 1)	−0.2293	0.1555
Tenure	−0.0393^b^, **	0.0129
Perceived health	−0.0295	0.0675
Independent variables:		
Occupational stress (X)	0.4084^a^, *	0.1773
Presenteeism (M)	0.2895^a^, *	0.1282
POS (V)	−0.2750^b^, **	0.0991
Presenteeism x POS	−0.1819^a^, *	0.0902
*R* ^2^	0.2453*, ***	

Conditional indirect effect of OS (X) on turnover intention (Y) through presenteeism (M) at the levels of POS (V)^†^, ^b^		
POS	Effect (Boot SE)	Boot 95% CI
*−1 *SD* (−0.8016); Low*	0.2109 (0.0749)	0.0673; 0.3684
Medium (0.0000)	0.1403 (0.0616)	0.0233; 0.2697
*+1 *SD* (0.8016); High*	0.0696 (0.0626)	−0.0552; 0.1928

Abbreviation: SE, standard error.

^a^ Unstandardized coefficients and standard errors are reported; Direct and total effects.

^b^ Bootstrap sample size = 5000.

* *P* < .05.

** *P* < .01.

*** *P* < .001 (two‐tailed test).

Following Preacher et al’s (2007) suggestion, we operationalized the high and low levels of POS as one standard deviation above (0.8016) and below (−0.8016) the mean score, respectively. Table 3 also presents the estimates, standard errors, and significance value of the conditional indirect effects for OS across low, medium, and high levels of POS. The relationship was significant and positive when POS was low (−1 SD; 0.2109, CI = [0.07, 0.37]) or equal to its mean (0.1403, CI = [0.02, 0.27]), but not significant when POS was high (+1 SD; 0.0696, CI = [−0.06, 0.20]). Thus, the indirect conditional effect of OS on turnover intention through presenteeism depended on the levels of POS among OTs. The mediating effect of presenteeism in the positive relationship between OS and turnover intention was proved to be weaker for OTs who reported a higher level of POS. These results fully support Hypothesis 3. The bottom part of Table 3 reports the conditional indirect effects of OS on turnover intention through presenteeism across the levels of POS.

## 4 DISCUSSION

This research aimed to provide managers with insights into the mechanism of presenteeism; in particular, the way it affected employees’ intention to leave the organization. We analyzed the role of presenteeism as a mediator in the relationship between OS and turnover intention, and then explored the attenuating effect of POS on the positive presenteeism‐turnover intention relationship among OTs in Korea.

We found that OS among OTs is likely to prompt them to work while ill and such unproductive behaviors lead to turnover intention. These results support the prior studies that OS is a major predictor of presenteeism,^40^ with a significant and positive effect, and that presenteeism causes employees to underperform, which leads to detachment from, dissatisfaction with, and less commitment toward the organization,^41^ which in turn increase employee turnover intention. We also found that presenteeism played a mediating role in the relationship between OS and turnover intention. This indicates that OTs with OS are prone to possess the intention to leave the organization, if they engage in presenteeism. This finding was also supported by the bootstrapping results. High OS led to higher presenteeism (0.4845, *P* = .0000), which in turn led to higher turnover intention (0.3329, *P* = .0107). Thus, this indirect effect (0.1613) was derived by multiplying these two effects (0.4845 x 0.3329). The proportion of the mediated effect of OS on turnover intention by presenteeism could be calculated as 19.7% ((0.1613/(0.6556+0.1613))*100). These findings highlight the need to alleviate OS to decrease presenteeism. Managers should thus design and implement interventions for reducing and preventing OS.

Our results also confirmed the moderating role of POS in the causal relationship between presenteeism and turnover intention. The extent of the effect of presenteeism on turnover intention moderated by POS could be calculated as 62.8% ((0.1819/0.2895)*100). This finding validates empirical evidence indicating that POS may constrain OTs from developing the intention to leave, as POS is related to outcomes favorable to employees and is negatively related to turnover intention.^26^ Hence, POS plays a buffering role in mitigating the positive relationship between presenteeism and turnover intention. Consistent with an inducement‐contributions theory of voluntary turnover,^42^ we argue that the employee's willingness to engage in the organization depends on the difference between the inducements granted by the organization and the contributions anticipated of the employee. An organization that provides support may be perceived as offering greater inducement and thus may build a sense of liability in the employee to repay the organization. OTs who perceive greater inducements would be less likely to desire to leave the organization.

We also found that the indirect effect of OS on turnover intention through presenteeism was conditional, and depended upon the level of POS. This indirect effect emerged significantly only when OTs perceived low to medium levels of POS. POS is formed on the basis of an employee's entire relation with an organization, and thus is less vulnerable to change. Once employees appreciate that their organizations are committed to them, they develop affective commitment toward their organizations.^25^ Moreover, an employee who regards an employer as less supportive would be more likely to seek employment elsewhere. The employees’ perceptions of support from the organization would be the best predictors of their intention to stay or leave.

We observed notable findings related to the demographic characteristics of the participants. Female OTs, working in an occupation segregated by gender and in a country with well‐established gender stereotypes, often experience various indirect and direct forms of discriminations in terms of roles and expectations.^43^ Therefore, they are likely to experience higher OS. However, they still endeavor to comply with the conflicting demands from home and work, particularly OS due to the nature of work, which increases the likelihood of presenteeism^34^ and thus turnover intention. Moreover, OTs with longer tenures reported a negative relation with turnover intention. In contrast to the work culture in Western countries, the workplace climate in Korea is predominantly “collectivist”,^44^ wherein people favor the pursuit of group interests over personal gains. OTs with longer tenures of professional service may be more likely to remain in the organization because of this collectivistic culture. Consistent with past findings,^6^, ^34^ the perception of individual health condition was another major predictor of presenteeism. Furthermore, the results revealed that OTs working as permanent employees reported higher presenteeism and lower POS than non‐permanent OTs. The social strain from the responsibility and role demands in a permanent position may increase the risk of presenteeism compared with the relatively free non‐permanent positions. Conversely, OTs in temporary or contractual positions may feel less responsible on the job and may have the tendency to believe they, with their government‐warranted professional license, are marketable.

Our study has a number of implications for future research and practice. In terms of the theoretical perspective, the present study extends the current understanding of presenteeism in the OT field. First, our conceptual framework makes a novel contribution to the presenteeism literature by applying JD‐R theory, transactional theory of stress, and COR theory as explanatory concepts to examine the causal relation of OS on presenteeism. Second, as studies on the mediational relation of presenteeism are scarce, our work contributes to the research on the mediational role of presenteeism between OS and employees’ attitudinal variables, such as turnover intention. Third, from the perspective of transactional theory of stress and COR theory, presenteeism as a coping strategy may provide a theoretical ground for a deeper understanding of the proposed relations. Therefore, our work suggests the emergence of OS through presenteeism on the resultant turnover intention.

As for the practical perspectives, organizations planning to develop HR programs and policies for the higher retention of OTs can use this framework to implement HR interventions to minimize OS and presenteeism. For example, to prevent presenteeism, organizations may employ health counselors to consult regularly with OTs regarding health and stress conditions at work. Organizations focusing on lowering actual turnover should also be aware of presenteeism and thereby redesign job descriptions and responsibilities as a measures to make OTs feel more cared for and supported. Hence, our findings alert managers to the need to manage OS and be cautious about the consequences of presenteeism. We also encourage them to increase POS and reduce OS, to decrease the risk of actual turnover.

## 5 LIMITATIONS OF THE STUDY

The current findings should be considered in light of the following limitations. First, we defined OS as a predictor of presenteeism among OTs. However, many studies have indicated that other work‐related factors should be evaluated as predictors for presenteeism; for example, leadership style, organizational culture, and socio‐demographic variables could potentially influence presenteeism as well. Second, we only discussed the associations between the variables of interest, ruling out unobserved attributes, such as personality traits, for which were not controlled for. Third, as we collected cross‐sectional data, which may limit the justifications of the causal order among our tested variables, we recommend a longitudinal research design with at least two waves to confirm the causality of our hypotheses. Fourth, although we collected data by visiting each health care institute and controlled a few of the covariates in our analyses, we could not guarantee that we have avoided the issue of potential social desirability in OT participants’ responses to the questionnaire items. Fifth, we were also unable to identify the distinctions of OTs working in public and private‐owned health care facilities. Caution needs to be exercised in generalizing the interpretation of the study results to the global OT industry. Hence, these areas warrant further investigation in future research.

## 6 CONCLUSION

The potential adverse effects of attendance dynamics such as presenteeism have not received adequate attention in the workplace setting. In this regard, our findings provided fresh insights into the dynamics of presenteeism by exploring the mediating relationships between OS and turnover intention, and by investigating the potential moderating role of POS in shaping the link between presenteeism and turnover intention in the OT workforce in Korea. Our findings may help managers in health care institutions better understand the predictors and consequences of presenteeism and, in particular, the mechanism of the conditional effects of OS through which presenteeism affects subsequent attitudinal behaviors. Our study represents the first attempt, to the best of our knowledge, to investigate the role of a psychosocial factor, ie, POS, in minimizing turnover intention affected by presenteeism among OTs in Korea. We hope that our findings offer a preliminary empirical basis for managers to recognize the importance of managing POS and reducing OS and presenteeism, and thus, turnover intention, among OTs.

## DISCLOSURE

*Approval of the research protocol:* Approval to conduct the study was obtained from the IRB committee at Gwangju University in Korea (approval #: 201906‐HR‐004‐02). *Informed consent* was obtained from each participant. *Registry and the registration no. of the study:* N/A. *Animal studies:* N/A.

## CONFLICT OF INTEREST

The authors declare no potential conflicts of interest with respect to the research, authorship, and/or publication of this article.

## AUTHOR CONTRIBUTIONS

All the authors were involved in developing the research questions and study design. The two authors agreed on the methodology for the analyses. Then, the first author analyzed the data and discussed the findings with the second author. The first author drafted the methods and findings sections, while the second author drafted the intro and discussion. After all the authors provided the inputs on the draft of the manuscript, the second author finalized it for submission.
